# The development of information infrastructure and technological capabilities used to manage social care and address quality in primary care settings

**DOI:** 10.1186/s12913-025-12821-7

**Published:** 2025-05-09

**Authors:** Anthony M. Provenzano, Faiyaz Syed, Jodyn E. Platt, Gretchen A. Piatt, Mark S. Ackerman, Ayse Buyuktur, Michael S. Klinkman

**Affiliations:** 1 Department of Learning Health Sciences, Medical School, University of Michigan, Ann Arbor, MI USA; 2 Michigan Primary Care Association, Lansing, MI USA; 3 Department of Electrical Engineering and Computer Science, School of Information, University of Michigan, Ann Arbor, MI USA; 4 Michigan Institute for Clinical and Health Research, University of Michigan, Ann Arbor, MI USA; 5 Department of Family Medicine, Medical School, University of Michigan, Ann Arbor, MI USA

**Keywords:** Primary care, Information technology, Social determinants of health, Technological capabilities, Social care, Quality improvement, Community health centers

## Abstract

**Background:**

With new payment systems to prompt more sophisticated data activities, primary care practices are developing technological capabilities to manage patient care and information. One burgeoning capability is the collection of social determinants of health (SDOH) data and using that information to provide social care. This study describes the information infrastructure and technological capabilities developed by community health centers (CHCs) and examines the factors influencing SDOH data integration and management in primary care practice. It offers health care leaders insights and strategies to build capacity for managing social care and quality.

**Methods:**

An observational design was used to examine the technological capabilities of CHCs in Michigan via a practice survey, and factors related to developing information infrastructure were qualitatively explored. The practice survey, semi-structured interviews, and national health center data were analyzed. Sociotechnical systems and organizational theories were used to develop the survey and interview guide. A sample of Michigan CHCs (*n* = 15) was recruited for the study. The practice survey was administered to CHC leaders, clinicians, and staff (*n* = 27). Semi-structured interviews (*n* = 25) were then conducted to explore infrastructural, organizational, and technological factors associated with managing social care and information.

**Results:**

Michigan CHCs developed capabilities to exchange patient information with state and local partners. Data were typically shared with maternal and infant health (*n* = 5, 33.3%), mental health (*n* = 5, 33.3%), substance use (*n* = 6, 40%), domestic violence (*n* = 6, 40%), and food assistance (*n* = 6, 40%) providers, but CHCs did not develop the same capabilities with all social services examined. The interviews revealed that CHCs leveraged health care and government investments in information technology (IT) as a strategy to share data and address quality. The survey results revealed that CHCs developed the ability to use SDOH data to manage population health and provide value-based care.

**Conclusions:**

IT used to manage social care and address quality is necessary but insufficient in primary care settings. The technological capabilities developed to integrate SDOH data into practice and exchange health information support critical infrastructure and learning opportunities to improve care, quality, and outcomes.

## Introduction

Following the passage of the Health Information Technology for Economic and Clinical Health (HITECH) Act of 2009, electronic health records (EHR) were widely adopted by hospitals and medical practices throughout the United States [1]. While HITECH supported the widespread adoption and meaningful use of EHR systems, the information infrastructure it created was not designed to effectively manage social care and address quality [2]. The Medicare Access and CHIP Reauthorization Act of 2015 changed the landscape by establishing data-driven payments on the basis of clinical performance and quality measures [3]. This shift from volume to value has accelerated the adoption of information technology (IT), modernizing data management and health care practices. However, competing IT vendors, incompatible data systems, and a lack of incentives provided to all professionals involved in sharing health information remain barriers to improving care, quality, and outcomes [4].

The 21st Century Cures Act addressed some infrastructural and technical challenges, creating policies and programs that certify health IT and establish common data standards. New rules for EHR system application programming interfacing (APIs) and case reporting provide the data governance necessary to share health information more broadly [5]. With new policy priorities and payment systems to prompt more sophisticated data activities (e.g., information exchange, risk-adjusted payments), primary care practices are investing in EHR systems and other IT to better manage patient care and information [6]. The investments being made in IT infrastructure support the development of technological capabilities, generating immense opportunities for quality improvement and learning to enhance care, effectiveness, and efficiency [7].

One burgeoning technological capability is the collection of social determinants of health (SDOH) data (e.g., housing, food insecurity) and the use of that information to provide social care (e.g., housing services, food assistance) [8]. Health care organizations, including primary care practices, are increasingly documenting SDOH data in EHRs and incorporating that information into staffing workflows [9]. However, studies systematically examining SDOH data integration and care management are needed to guide this work. To the best of our knowledge, this is the first study of its kind to examine local information infrastructure and partnerships developed to manage social care and address quality across a statewide network of community health centers.

Nationally, community health centers (CHCs) provide primary and preventive care to more than 12,000 urban and rural communities. CHCs are leading efforts to integrate social care into practice [10], as evident by the adoption of SDOH data collection standards [11] throughout its network of 1,370 centers [12]. Collectively serving over 30 million patients, annually, most CHCs screen for social risks (71%) [13], treat, or refer patients to government programs and community services. Coordinated efforts are also underway to support CHCs in adopting EHR-embedded screening and data analytic tools via their involvement in practice-based networks [14]. Where IT adoption and SDOH data integration are most ubiquitous, CHCs leverage public and private investments in health information exchanges to share patient data with care partners.

The success of CHCs in establishing sustainable practices for managing social care and improving quality using SDOH data offers insight and provides a setting in which to examine the barriers and facilitators of developing infrastructure to deliver high-quality care. In this study, an academic-community research partnership guided the investigation of services and IT adopted by Michigan CHCs and local organizations to provide medical and social care. The purpose of the study was twofold: (1) offer public officials and health care leaders a better understanding of the partnerships and data activities developed to manage patient care and population health and (2) present primary care practices with strategies for building capacity to support social care and quality improvement activities.

The study describes the technological capabilities developed by Michigan CHCs and reveals factors influencing SDOH data integration and care management. Investigators have drawn on sociotechnical theory to reveal human/organizational and technical elements of information infrastructure [15, 16]. A sociotechnical systems model was used to categorize data activities, shared IT, and partnership agreements (i.e., service, data use) developed to coordinate care and address quality [17]. The results were presented to answer the following research questions: What technological capabilities have CHCs developed to manage patient care and information? What are the barriers to developing capabilities that support SDOH data collection and use? What are the facilitators of developing information infrastructure used to coordinate care and address quality?

## Methods

### Study design

This is an observational study of information infrastructure and technological capabilities developed by CHCs. The study design was informed by the sequence of the quantitative and qualitative data collection and analyses. A practice survey, secondary data analysis, semi-structured interviews, and qualitative rapid analysis procedures were used in consecutive phases. Investigators defined technological capabilities as (a) integrating and managing SDOH data (i.e., digitally screening and tracking care needs/referrals); (b) achieving data interoperability with care partners (i.e., digitally exchanging data and sharing information systems); and (c) conducting data activities (i.e., using SDOH information for quality improvement, risk stratification, and risk adjusted payments).

### Recruitment and sampling procedures

Investigators identified and sampled participants from CHCs through an academic-community research partnership with the Michigan Primary Care Association and its statewide network of primary care practices in Michigan. The investigation was limited to Michigan CHCs (*N* = 40). The study was determined to be exempt from review by the University of Michigan Institutional Review Board. All CHCs in Michigan were eligible to participate in the study. The CHCs were recruited via a webinar in June 2021. Webinar attendees consisted of quality directors and chief operating officers from all 40 Michigan CHCs. Purposive sampling was used to correctly identify the appropriate employee to enroll in the study. Webinar attendees were invited to complete an IT practice survey or forward it to the person in their practice with knowledge of IT and partnerships used to manage patient care and information. Study participants completed the practice survey in July and August 2021. Investigators then contacted survey respondents to schedule follow-up interviews that occurred in September and October 2021. Gift cards ($25 each) were provided to the study participants for both the survey response and interview participation. Informed consent was obtained from each participant interviewed and surveyed.

### Quantitative data collection and analysis

#### Practice survey

The IT practice survey was created to examine the information infrastructure developed by CHCs to collect, track, exchange, and analyze patient data. The survey was designed by adapting a framework [18] based on Walter Leutz’s organizational theory [19]. A six-point scale was used to measure the technological capabilities: *collecting SDOH information* by using IT to digitally screen SDOH risks and record diagnoses; *managing SDOH data using information systems* to track care needs and referral status; *achieving data interoperability with partners* to share information systems and/or digitally exchange SDOH data; and *conducting data analytics and reporting* using information infrastructure to manage patient panels, inform quality improvement, and create risk stratification models for targeted interventions and/or for adjusting payments. The survey measures were developed through the academic–community research partnership and pilot tested with health centers. The full survey instrument is available in the author’s dissertation [20]. Descriptive statistics (*n*, %) were used to calculate the frequencies and percentages of the CHC technological capabilities.

#### Secondary data

Health Resources and Services Administration (HRSA) data were used to describe the clinical characteristics of health centers, explain clinic settings (urban vs. rural), and contextualize site-level differences related to IT used. A database was created to merge and store primary and secondary data. The database consisted of survey data collected and practice-level information obtained from HRSA’s Uniform Data System (UDS) [21]. Descriptive statistics were performed using the following variables from the 2021 UDS dataset: *practice size*, i.e., total patients, annual expenditures, per patient costs, *setting* (rural/urban), *patient demographics*, and *insurance coverage* information. ANOVA and chi-square tests were performed using UDS data to examine associations between the national, state, and study samples.

### Qualitative data collection and analysis

A semi-structured interview guide was created to explore infrastructural, organizational, and technological factors associated with developing data systems to manage care. Sittig and Singh’s sociotechnical systems model for studying IT adoption and use in adaptive healthcare settings informed the questions asked [17]. All 8 dimensions of their model — hardware and software; clinical content; people, workflow and communication; human‒computer interface; organizational policies and procedures; culture; external rules, regulations, and pressures; and system measurement and monitoring — were used to conceptualize the characteristics of the information infrastructure and community partnerships developed. Leutz’s organizational theory [19] was also used to inform the interview guide and help describe the complexity of local governance and partnership agreements, e.g., community priorities, shared IT capabilities, and data policies. Study participants were asked about the workforce and technological challenges in providing social care and to describe the barriers and facilitators of developing care management and information infrastructure. The 22-question interview guide was developed, iterated, and tested through the academic–community research partnership. The complete interview guide is available in the author’s dissertation [20].

A qualitative rapid assessment process (RAP) was used to develop a data extraction template tool based on the sociotechnical model described. RAP is an intensive, team-based qualitative inquiry that uses data triangulation, iterative analysis, and additional data collection to quickly develop an understanding of a situation, setting, or phenomenon from an insider’s perspective [22]. RAP is a demonstrated and efficient method for time-sensitive health services research and is used in evaluations of applied clinical informatics across settings (e.g., hospitals, primary care) [23]. The data extraction template tool was tested by the investigators for consistency and reliability before transcript coding commenced. The coding occurred until data saturation was achieved, and disconfirming data were no longer identified. Data matrices were derived from the extraction template tool and used to capture coded information regarding sociotechnical domains from the transcripts. Domain and subdomain summary profiles were then created to analyze the multilevel factors, barriers, and facilitators associated with site, setting, and service differences. A thematic analysis was performed to identify sociotechnical factors and themes related to partnership development and sharing information with care partners.

## Results

Table 1 presents the clinical characteristics of CHCs. Patient age and sex were similar among national, state, and sample groups. Although, there were significant differences (*p*-value = 0.0075) in race/ethnicity among CHCs nationally (57.1%), statewide (58%), and in the sample (63%) for white, non-Hispanic patients. Like most CHCs in the United States, Michigan CHCs predominantly served Medicaid beneficiaries (51.6%) and the uninsured (10.8%). Investigators found a significant difference (*p*-value = 0.0349) examining practice setting with more rural CHCs in the study sample (53.3%), when compared statewide (35%) and nationally (42%). A significant difference (*p*-value < 0.001) was identified between national (6.9%), state (8.4%), and sample (10.7%) groups among CHCs serving patients living 200% below the poverty level. Table 2 list survey respondent titles by position type and practice setting. Twenty-seven leaders, clinicians, and staff from fifteen Michigan CHCs (*n* = 15) were surveyed (37.5% response rate). Eight practices had two or more survey respondents, and nine practices had one respondent. There were 6 executives, 6 quality staff, 3 practice managers, 4 program staff, 7 care managers, and 1 IT coordinator who responded to the survey.

**Table 1:** Clinical characteristics

Variables	Nationally *N* (%)	Statewide *n* (%)	Sample *n* (%)	*p*-value for difference
Patients	30,193,278	656,456	311,309	
Age				
Children < 18 years old	8,584,984 (28.4)	170,143 (25.9)	91,097 (29.3)	0.9896
Adults 18–64	18,250,848 (60.7)	408,023 (62.2)	181,614 (58.5)	
Older Adults 65+	3,278,358 (10.9)	75,950 (11.9)	38,012 (12.2)	
Sex				
Female	17,314,000 (57.3)	373,767 (56.9)	174,458 (56.0)	0.9865
Male	12,879,278 (42.7)	282,779 (43.1)	136,851 (44.0)	
Race/Ethnicity				
White non-Hispanic	17,409,098 (57.7)	380,534 (58.0)	196,142 (63.0)	0.0075^*^
Black non-Hispanic	5,428,337 (18.0)	171,588 (26.1)	59,548 (19.1)	
Hispanic/Latino	6,309,905 (20.9)	33,391 (5.1)	20,661 (6.6)	
Asian	1,045,938 (3.5)	8,428 (1.3)	3,464 (1.1)	
Income status				
100% of poverty level	13,379,543 (44.3)	282,062 (39.3)	124,681 (40.1)	< 0.001^*^
200% of poverty level	2,083,021 (6.9)	55,290 (8.4)	28,793 (10.7)	< 0.001^*^
Insurance type				
Medicaid/CHIP	14,465,291 (48.0)	338,790 (51.6)	156,916 (50.4)	0.4481
Medicare	3,211,698 (10.6)	95,563 (14.6)	46,260 (15.0)	
Other third party	6,131,304 (20.5)	149,959 (23.0)	74,392 (23.9)	
Uninsured	6,136,642 (20.9)	70,891 (10.8)	33,461 (10.7)	
No. of CHCs	1,370	40	15	
Setting				
Rural	574 (42.0)	14 (35.0)	8 (53.3)	0.0349^*^
Urban	796 (58.0)	26 (65.0)	7 (46.7)	
Practice size mean, sd (range)				
No. of patients	21,991 ± 27,153 (131–247,428)	16,592 ± 13,013 (1,763 − 54,383)	20,754 ± 13,961 (1,763 − 54,383)	< 0.001^*^
Annual expenditures	26,797,698 ± 39,836,466 (898,037–772,495,904)	19,269,587 ± 15,699,587 (3,140,160 − 73,746,994)	25,709,699 ± 17,692,745 (3,402,781 − 73,746,994)	< 0.001^*^
Per patient costs	1,398 ± 1,423 (144 − 39,739)	1,283 ± 607 (551-3,701)	1,306 ± 244 (836-1,930)	< 0.001^*^

^*^p-value is less than 0.05

**Table 2:** Study participant title by position type, practice setting, and data collection method used

Position Type	Survey and Interview (*n* = 19)	Survey Only (*n* = 8)	Interview Only (*n* = 6)
Executives	Chief Medical Officer Chief Operating Officer Executive Director Clinical Operations Director^*^	Chief Analytics Officer Chief Operating Officer	Chief of Behavioral Health And Integrated Services^*^
Quality Staff	Quality Improvement Director^*^ (2) Quality and Informatics Director Quality Manager^*^ Quality Support Specialist	Director of Quality^*^	Director of Quality Improvement Quality RN
Practice Managers	Clinic Manager Manager of Patient Services^*^	Enabling Services Manager^*^	
Program Staff	Youth and Legal Program Director Behavioral Health and Social Work Supervisor Population Health Supervisor^*^	Substance Use Program Manager	
Care Managers	RN Care Coordinator Manager^*^ Population Health Manager^*^ Public Health Worker Community Health Worker (2)	Community Health Worker Supervisor* Community Health Worker*	
Outreach Staff			Resource Specialist Outreach Supervisor^*^
IT Support		IT Coordinator	EHR Support Manager^*^

*RN* Registered Nurse, *IT* Information Technology, *EHR* Electronic Health Record

^*^Rural Practices

### SDOH data integration and management

**Fig. 1:**
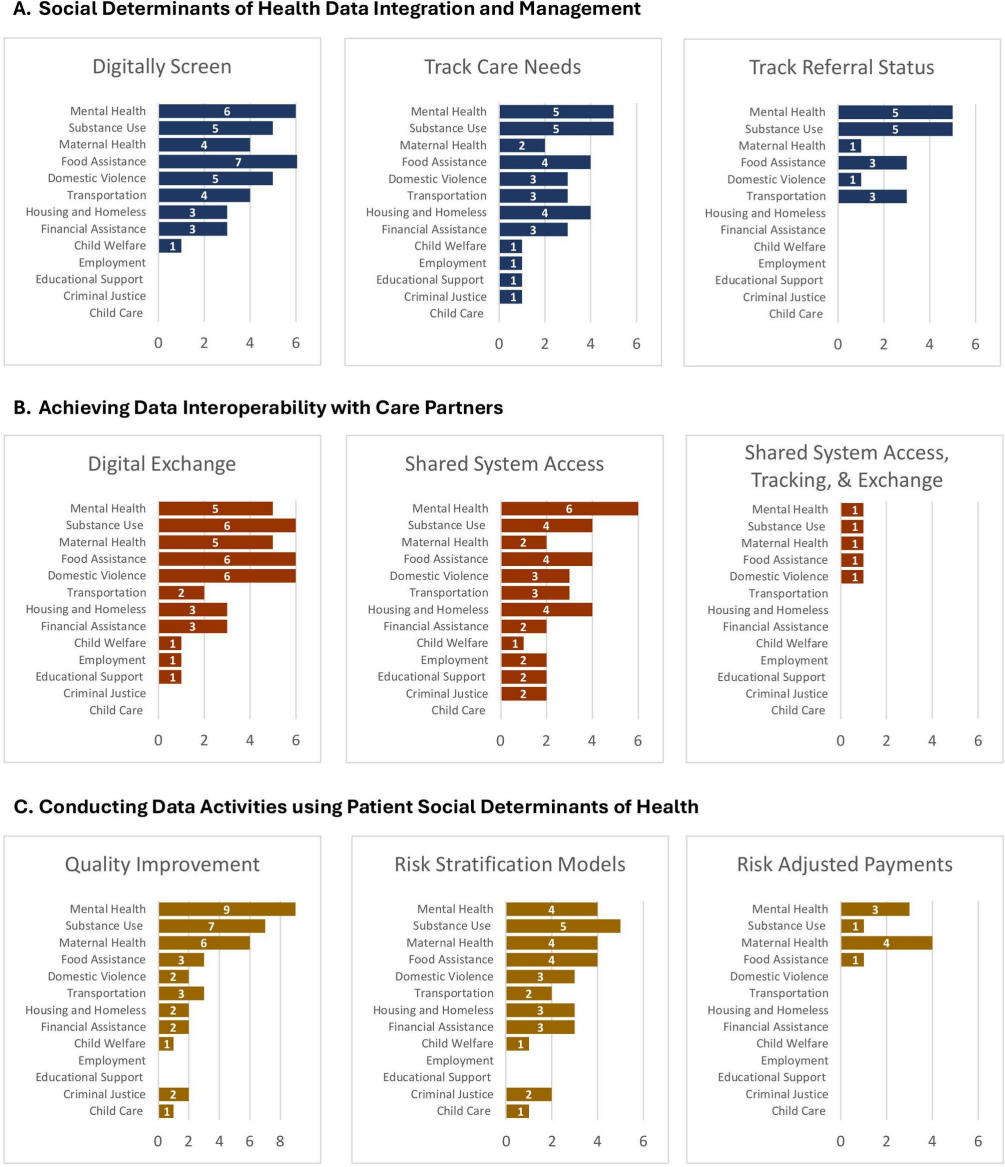
Technological capabilities of community health centers in Michigan (*n* = 15)

Figure 1 displays the survey results. Figure 1A *Social Determinants of Health Data Integration and Management* presents the CHC technological capabilities developed to collect and manage SDOH data. One-third of Michigan CHCs sampled (*n* = 5; 33.3%) digitally screened risks, tracked care needs and referred patients to behavioral health services. Approximately 27% of the CHCs sampled (*n* = 4; 26.7%) digitally screened maternal and infant health risks, and only one health center tracked referral status. Tracking referrals were often not needed because many Michigan CHCs sampled accessed health department information systems used to manage shared patients. For social care data integration and management, the investigators found that nearly half of the CHCs sampled digitally screened for food assistance (*n* = 7; 46.7%), one-third for domestic violence (*n* = 5; 33.3%), and approximately one-fourth for transportation needs (*n* = 4; 26.7%). Some of the CHCs sampled tracked referrals for food assistance (*n* = 3; 20%) and housing services (*n* = 3; 20%).

### Achieving data interoperability with partner organizations

Figure 1B *Achieving Data Interoperability with Care Partners* presents capabilities developed by Michigan CHCs to digitally exchange patient data or share information system access with government and community organizations. One-third of CHCs in the sample exchanged patient information with maternal and infant health (*n* = 5; 33.3%), mental health (*n* = 5; 33.3%), and substance use disorder (*n* = 6; 40%) partners. The CHCs in our survey accessed local health department, state community mental health, and public assistance (e.g., TANF, Medicaid) data systems to manage shared clients. The CHCs also granted social care providers access to their own EHR systems to exchange patient information. Many CHCs surveyed electronically shared patient data with food assistance (*n* = 6; 40%) and domestic violence (*n* = 6; 40%) organizations. A few had established the same capability with housing (*n* = 3; 20%), financial assistance (*n* = 3; 20%), and transportation (*n* = 2; 13.3%) partners. Generally, the Michigan CHCs sampled did not achieve the same degree of data interoperability with social service providers because their partners lacked the necessary information infrastructure and technical expertise.

### Conducting data analytics and reporting using SDOH information

Figure 1C *Conducting Data Activities Using Patient Social Determinants of Health* presents the data used by CHCs in risk stratification modeling, providing value-based care (i.e., creating risk-adjusted payments), and making quality improvements. One-third of CHCs (*n* = 5; 33.3%) sampled used substance use disorder data to conduct predictive analytics. Approximately 25% used maternal and infant health (*n* = 4; 26.7%), mental health (*n* = 4; 26.7%), and food assistance (*n* = 4; 26.7%) data to develop risk stratification models. A few CHCs created risk-adjusted payments using maternal and infant health (*n* = 4: 26.7%); mental health (*n* = 3; 20%); substance use disorder (*n* = 1; 6.7%); and food assistance (*n* = 1; 6.7%) data.

### Qualitative results

Follow-up interviews (*n* = 25) were conducted with survey respondents from 6 urban and 5 rural participating CHCs. Two or more interviews were conducted with the seven practices, and one interview with four practices. There were 5 executives, 7 quality staff, 2 practice managers, 3 program staff, 5 care managers, 2 outreach staff, and 1 IT support staff interviewed. Table 2 lists the titles of the interviewees by position type and practice setting. The results from an analysis of interviews revealed infrastructural, organizational, and technological factors associated with managing medical and social care. Barriers and facilitators of developing information infrastructure and technological capabilities used to integrate, manage, and exchange SDOH data are described.

### Barriers to developing technological capabilities

Table 3 summarizes barriers to developing the ability to manage patient care and information.

**Table 3:** Barriers to developing technological capabilities used to manage patient care and information

Factors	Barriers	Quotes
Infrastructural	Limited shared vision throughout communities	“Getting everybody on the same page in the community to respond to a shared spot, a shared database is a problem.” *Practice Manager*
		“There is poor accountability in the community regarding service referrals.” *Care Manager*
	High staff turnover and shortages	“With [partner] staff changes, keeping workflow processes running well in itself is a challenge.” *Quality Director*
Technological	Overreliance on manual processes	“We don’t really have that kind of relationship with our partners generally speaking, where we use technology for referrals.” *Care Supervisor*
		“There are a lot of manual processes in terms of sharing [patient information].” *Practice Manager*
	Excessive amounts of data	“The excess of patient data available makes tracking and sharing relevant information with partners difficult, and oftentimes information is not readily or easily accessible to our providers and staff.” *Community Health Worker*
Organizational	Multiple reporting platforms and IT limitations presented challenges for data integration	“…double-charting due to multiple reporting guidelines and ill-matched information systems present challenges.” *Quality Director*
	Leadership turnover disrupted workflows and staff development	“It is a challenge when we’ve had a change in leadership, change in multiple support staff, change in providers, and don’t have enough bandwidth from our CMO to make sure each provider is onboarded and trained, and making referrals in the same way.” *Quality Director*
Patient-level	Technology is not reaching all communities and vulnerable populations	“Some patients have a really tough time connecting to the internet or have no WIFI access at all.” *Quality Director*
		“Homeless populations lack permanent addresses, and the internet does not reach remote communities, making Telehealth difficult.” *Care Manager*
		“Cell phone service shut-offs limit providers’ referral and follow-up capacity.” *Care Manager*

*CMO* Chief Medical Officer

#### Infrastructural barriers

Workforce capacity issues impeded IT capability development. Staffing shortages across IT and data analytic teams were frequently reported during interviews with quality directors and managers. High staff turnover and shortages necessitated frequent training and additional onboarding to introduce IT practices, data policies, and processes: a quality director stated, “with [partner] staff changes, keeping workflow processes running well in itself is a challenge.” CHC leadership and staff also described limited shared vision throughout communities as a barrier to collectively developing local information infrastructure. A practice manager stated, “Getting everybody on the same page in the community to respond to a shared spot, a shared database…” had hindered progress. A care manager further explained, “There is poor accountability in the community regarding service referrals,” stressing workforce issues and a lack of shared vision for ensuring care continuity.

#### Technological barriers

During the interviews, CHC directors and care staff discussed overreliance on partner manual processes and incompatible EHR systems as barriers to developing capabilities to become data interoperable. A practice manager stated, “There are a lot of manual processes in terms of sharing [health information].” Additionally, a care supervisor said, “We do not really have that kind of relationship with our partners generally speaking, where we use technology for referrals.” A quality director also described efforts to adopt software as a community strategy to “bridge the gap” between incompatible care management data systems.

#### Organizational barriers

During the interviews, quality directors and care managers discussed barriers to integrating SDOH data into care and information systems. Preventive screenings, data storage, and referrals were often paper-based, and multiple reporting platforms presented challenges with data and care management. A quality director explained, “When providing treatment plans or resources for patients, the responsibility to follow up typically falls to the patient, meaning referrals often require a direct request for continuity of support by the patient.” A care manager echoed, “I mean, we give them the resources, but there’s not an extra follow-up.” A quality director discussed how cumbersome their data integration processes were, “double-charting due to multiple mandatory reporting guidelines and ill-matched information systems present challenges.”

#### Patient-level barriers

During the interviews, CHC directors and managers revealed the technological challenges related to reaching patients and other vulnerable high-risk populations, particularly those from low-income and rural communities: a care manager said, “Homeless populations lack permanent addresses, and the internet does not reach remote communities, making telehealth difficult.” A quality director also stated, “Some patients have a really tough time connecting to the internet or have no WIFI access at all.” Another care manager explained the root of the issue as a financial matter: “Cell phone service shut-offs limit providers’ referral and follow-up capacity.”

### Facilitators of developing information infrastructure

Table 4 summarizes the facilitators of developing IT to coordinate care and address quality.

**Table 4:** Facilitators of developing information infrastructure used to coordinate care and address quality

Technological Factors	Facilitators	Quotes
SDOH Data Infrastructure and Standards	IT use supported SDOH data integration, care management, and quality improvement	“We can see the chart, whether that particular need had been addressed or if there’s additional follow-up and work that needs to be done.” *Care Manager*
		“Z-code encounter reasons provide data necessary to enhance quality improvement initiatives.” *Quality Director*
		“Risk stratification is made possible through the EHR system, using data such vital health indicators, patient lifestyle, and medical history.” *Care Manager*
Data Policies, Rules, and Procedures	EHR system use helped with implementation of internal data policies and activities	“Some notes can be marked sensitive, and then there’s some patients that you’re not able to access. You have to request access.” *Social Worker*
		“Our internal data protections prevent sharing patient information without first receiving patient consent.” *Practice Manager*
Existing Information Infrastructure	Local IT infrastructure provided access to shared patients	“We have access to EHRs of all the local hospitals in our area, so the care connectors will actually go in and pull reports of patients that have in the ED there, and they do outreach calls. See if they need follow-up and try to decrease ED utilization.” *Quality Manager*
		“We exchange data with the health department… providing biometric information on patients with chronic disease.” *Program Manager*
External Policies and Regulations	Quality measures supported new care management and monitoring activities	“We always work with the health plans on HEDIS required reporting of that information that helps drive revenue back to us. And it helps us to hire additional people to monitor for quality.” *Executive Director*
New Technology	Cell phones and telehealth connected patients to care	“Most people can follow a link on their phones. And we learned this during the pandemic when people were doing medical visits.” *Care Manager*
		“Patients can just snap a picture with their phone and upload requested documentation right into their case.” *Care Manager*

*SDOH* Social Determinants of Health, *EHR* Electronic Health Record, *IT* Information Technology, *ED* Emergency Department, *HEDIS* Healthcare Effectiveness Data and Information Set

#### SDOH data infrastructure and standards

During the interviews, quality directors and care managers discussed the advantages of using IT to integrate SDOH data, manage care, and address quality. Specific Z-codes (Z55– Z65) were used to identify and track patient needs related to the SDOH: a care manager said, “We can see the chart, whether that particular need had been addressed or if there’s additional follow-up and work that needs to be done.” A quality director stated, “Z-code encounter reasons provide the data necessary to enhance quality improvement initiatives.” A care manager described the capabilities developed using data infrastructure: “Risk stratification is made possible through the EHR system, using data such as vital health indicators, patient lifestyle, and medical history.”

#### Data policies, rules, and procedures

Interviews with CHC managers and care staff revealed that using information systems supported implementation of internal data policies and activities. CHCs often used EHR systems to restrict sensitive data to a “need to know” basis and controlled staff access using different workflows and access permissions. For example, care managers were to document patient consents in EHR systems before data permissions were authorized to share information with partners: a social worker supervisor said, “Some notes can be marked sensitive, and then there’s some patients who you’re not able to access. You have to request access.” A practice manager also stated, “Our internal data protections prevent the sharing of patient information without first receiving patient consent.”

#### Existing information infrastructure

During the interviews, care and quality managers discussed the local IT infrastructure used to obtain access to shared patients: a quality manager stated, “We have access to EHRs of all the local hospitals in our area, so the care connectors will actually go in and pull reports on our patients who have been in the ED there, and they do outreach calls. See if they need follow-up and try to decrease ED utilization.” A program manager from another practice said, “We exchange data with the health department…. providing biometric information for patients with chronic diseases.”

#### External policies and regulations

During the interviews, CHC executives discussed public and private payer incentives for care management and quality activities. An executive officer from an urban health center stated, “So obviously social determinants of health are a cost driver, so an expense. In addition, if we can eliminate those and maybe improve the person’s health. We would reduce the cost overall…we always work with the health plans on any HEDIS [Healthcare Effectiveness Data and Information Set] required reporting of that information that helps drive revenue back to us. In addition, it helps us hire additional people to monitor for quality.” Another executive officer from a rural practice described available payer incentives through practice-based networks: “MPCA [Michigan Primary Care Association] finances CHWs [Community Health Workers] through the patient navigator grant.”

#### New technology

CHC managers discussed capabilities prompted by the COVID-19 pandemic that enabled better patient care. With increased health care demands and rapidly evolving conditions, health center clinicians and staff had achieved greater levels of technological fluency and workplace adaptability. Pandemic restrictions gave rise to virtual appointments, digital medical documentation, and enhanced telecommunications that changed staffing workflows and care practices. A clinic manager described how workflow changes streamlined referrals: “Now, we have a resource department and logs to track things.” A care manager explained workflows using new technology: “Most people can follow a link. In addition, we learned this during the pandemic when people were doing medical visits on their phones.” Another care manager said, “Patients can just snap a picture with their phone and upload requested documentation right into their case.”

## Discussion

The study revealed that Michigan CHCs developed IT capabilities to manage care, quality, and population health using SDOH data and other medical information obtained from patients and their partners. Achieving data interoperability with state and local organizations provides CHCs with critical infrastructure and data practices used to coordinate care and exchange patient health information. Although Michigan CHCs were often challenged by complicated siloed sectors of care, fragmented communication, and limited resources [24, 25], study findings suggested that they were able to circumvent traditional referral processes (i.e., phone, email, fax) and overcome IT limitations by sharing data systems with their partners. Results demonstrated that authorizing EHR system access and other data permissions were used by Michigan CHCs to share patient information for meeting maternal health, behavioral health, and social care needs.

Consistent with the findings of Gold and colleagues, the results from this study offered evidence that IT was adopted to electronically screen SDOH risks and document care needs [9]. While this study also found Michigan CHCs generally developed IT capabilities to share patient information with public health and behavioral health sectors, rarely had they established similar capabilities with the complete range of social care providers examined. The study revealed that some Michigan CHCs digitally exchanged patient information with food assistance and domestic violence partners. Yet, most CHCs were not data interoperable with financial assistance, housing/homeless, transportation, child care, employment, and educational providers or had not developed the ability to share information with child welfare and criminal justice systems.

The qualitative results provide additional insight into the barriers Michigan CHCs encountered through partnerships with social service agencies. The findings suggested that overreliance on paper-based processes and incompatible care management systems thwarted IT development, data use, and the ability to digitally exchange patient information. The study results confirmed Onie and colleagues’ findings, suggesting that CHCs and their partners remained reliant on labor-intensive, unstructured and nonautomated types of data sharing because they often could not agree on common vendor platforms to exchange information [26].

Despite the technical challenges in communities throughout Michigan, CHCs established staffing workflows and data practices to better manage patient care and information. The study suggested that existing information infrastructure was key to CHCs establishing data policies [27] (e.g., ICD-10 Z-codes, access permissions) and achieving interoperability with partners. The investigation showed that Michigan CHCs documented SDOH information during patient visits and conducted annual SDOH screenings via office tablets, EHR portals, and email links. While Cole and colleagues’ national study revealed that nearly three-quarters of CHCs screened for SDOH risks [13], this study offered evidence that Michigan CHCs developed the same ability using digital collection methods. One-third of Michigan CHCs in this study, electronically screened for substance use disorder (33%) and domestic violence (33%) risks. Nearly half of Michigan CHCs sampled digitally screened for food assistance (47%), mental health needs (40%), and approximately one-quarter (27%) screened for transportation and maternal and infant health care.

The quantitative study results supported the qualitative findings by describing the types of partners with which CHCs shared information systems and whether they leveraged health care or government IT investments to develop these capabilities. The data confirmed that Michigan CHCs either shared state information systems with local health departments, regional mental health authorities, and the public assistance program, i.e., MI Bridges, or granted their partners access to their own EHR systems to collaboratively manage patient care and address community health needs. These IT strategies can be implemented by primary care practices to share patient information and better coordinate care with state and local partners. Although Michigan CHCs were able to overcome technological challenges of working across sectors, large scale policies remain imperative to establish a national social care information infrastructure.

This study’s design and interdisciplinary approach helped to describe the infrastructural, organizational, and sociotechnical factors related to SDOH data integration and management in primary care practice. The findings suggested that the IT infrastructure developed by Michigan CHCs helped control costs and support workforce development to address health disparities and to deliver high-quality care. The results demonstrated that some Michigan CHCs established the ability to use patient-reported and partner-generated SDOH data for managing practice panels and population health. Risk stratification models using SDOH and other health information were developed to identify social needs and target interventions for patients in chronic disease management programs. Risk-adjusted payments and pay-for-performance measures were often created with SDOH data to generate revenue for new care management and quality improvement activities. Like CHCs, primary care practices can negotiate new payments and quality measures with public and commercial health plans to better meet patient social needs and provide value-based care.

The study results offered evidence that Michigan CHCs developed information infrastructure and technological capabilities as a strategy to address care quality and outcomes. Local efforts to adopt IT and share patient data were leveraged by Michigan CHCs to improve care coordination and collective learning. Michigan CHCs capitalized on health care and government IT and new capabilities with state and local partners to deliver high-quality care. Functioning as distinctive learning health systems in communities throughout the United States, CHCs offer an exemplary model for developing IT infrastructure and technological capabilities. However, new national strategies and financial incentives are critical to support SDOH data integration and use in primary care settings.

### Limitations

There were significant differences in the clinical characteristics of CHCs nationally, statewide, and in the sample group that affected the study’s generalizability. Differences in practice size, setting (rural vs. urban), and patient income were attributed to smaller Michigan CHCs serving less diverse communities, when compared to other CHCs nationally. It is conceivable that CHCs participating in the study were treating patients with a greater need for social care. Furthermore, the scope of the study was limited to perspectives of Michigan CHCs, so it cannot account for potential regional variations or for the experiences of other types of organizations described in the study. The investigation was conducted during the COVID-19 pandemic, and IT research was not a high priority for CHCs, which influenced site recruitment and subject willingness to participate in the study. Although this affected sample size and generalizability, the interviews reached thematic saturation. In general, the interview respondents held other organizations and sectors of care responsible for the lack of local resources, services, and IT infrastructure. Future studies should identify the barriers and facilitators of managing social care and quality from the perspectives of government and community agencies. A greater understanding of the challenges and opportunities from their vantage point will better inform the policies and investments made to incentivize IT adoption for these critical partners.

## Conclusions

The technological capabilities developed using SDOH data and other patient information described in this study are necessary for managing social care and addressing quality. The findings highlight the advantages of leveraging local information infrastructure and how it may be used to improve care and health outcomes. The study results demonstrated that Michigan CHCs developed new technological capabilities to integrate maternal and infant health, behavioral health, and social care data into practice. By functioning as distinctive learning health systems in communities throughout Michigan, CHCs leveraged partnerships and IT investments to deliver and monitor high-quality care. This study offers health care leaders strategies and insights for developing sustainable care management and quality improvement activities using SDOH data.

## Data Availability

The datasets used and analyzed during the study are available from the corresponding author on reasonable request.
